# 328. Bacteremia in Patients Hospitalized with Covid-19 Disease, Risk Factors, Impact of immunomodulator Therapy, Role of Inflammatory Markers, Antibiotic Use, and Outcomes: A Single Center Retrospective Study

**DOI:** 10.1093/ofid/ofab466.530

**Published:** 2021-12-04

**Authors:** Aleena Zahra, Bettina C Fries, Bettina C Fries

**Affiliations:** 1 SUNY Stony Brook University Hospital, Stony Brook, New York; 2 Stony Brook University Hospital, Stony Brook, NY

## Abstract

**Background:**

Novel coronavirus 2019 (Covid19) caused by SARS-CoV2 can lead to significant morbidity and mortality. There is unclear association between Covid19 and bacteremia. Patient characteristics and outcomes are not well defined. This retrospective cohort study assessed this in patients with Covid19 and bacteremia.

**Methods:**

Patients with Covid-19 admitted to a tertiary care suburban academic medical center (UH) were assessed retrospectively by EMR chart review for co-morbidities, pre and in hospital factors, and outcomes as defined below. Bacteremias grouped into gram-negative or gram-positive with collation of each unique bacterial species (Table 1).

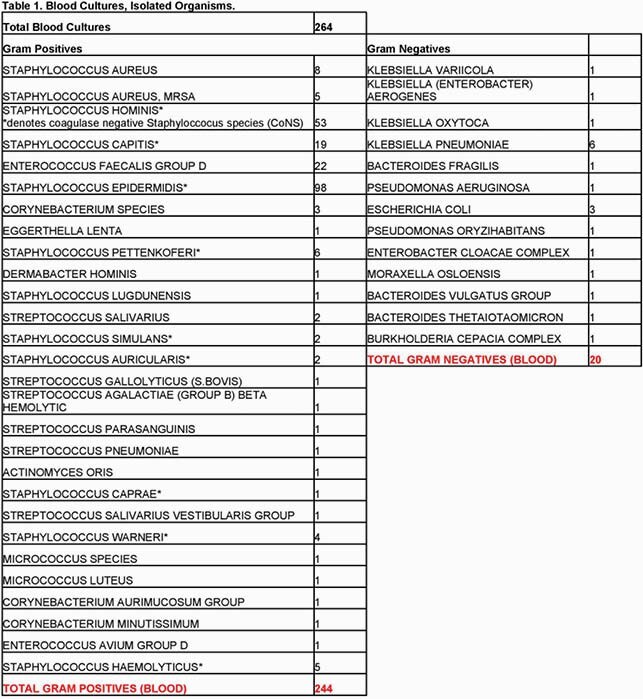

**Results:**

Total 1398 patients with Covid19 hospitalized at UH during local peak of pandemic of whom 238 (17.02%) developed 264 bacteremias with gram-positive (244, 92.4%) and gram-negative organisms (20, 7.57%). Relevant characteristics (Table 2) 53% with immunomodulator therapy (steroids/Tocilizumab), mean length of stay 21.04days (SEM ± 1.67) with day SARS-CoV2 PCR positivity -1.15days from hospitalization (SEM ± 0.49) and day initial bacteremia 6.38 (SEM ± 0.77), 55.4% required ICU admission, with 89% ICU admissions requiring mechanical ventilation. Most common co-morbidity (Figure 1 full list) Hypertension 56.3% followed by Obesity (BMI >30) 45.8% and CAD/CHF 40.3%. Laboratory parameters (Table 3)significant for average difference (date bacteremia- date admission) for Procalcitonin 4.15ng/mL (SEM ± 0.97, p-value 0.02), CRP -0.934mg/dL (SEM ± 0.95, p-value 0.32), WBC 7.027 K/uL (SEM ± 0.65, p-value < 0.005). These analyses excluded difference of 0 from hospital day 1 bacteremia. Average antibiotic number (1+ dose per antibiotic) 3.24 (SEM ± 0.16) and total C difficile cases 3 (1.26%). Mortality rate34.45%.

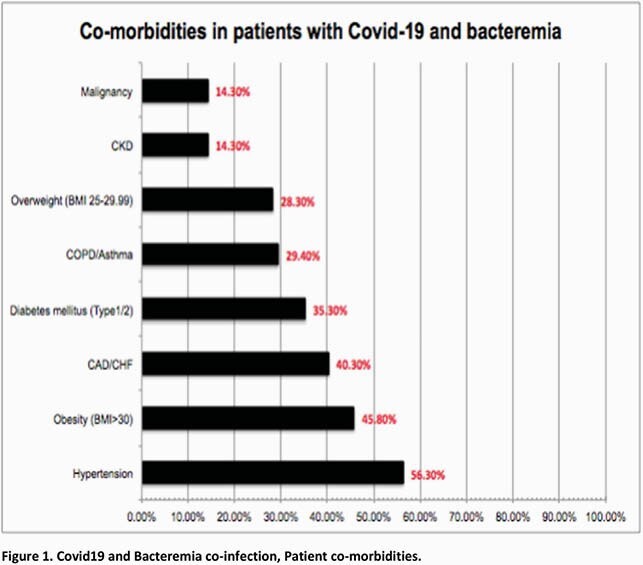

Relevant hospitalization characteristics, Covid19 and bacteremia co-infections

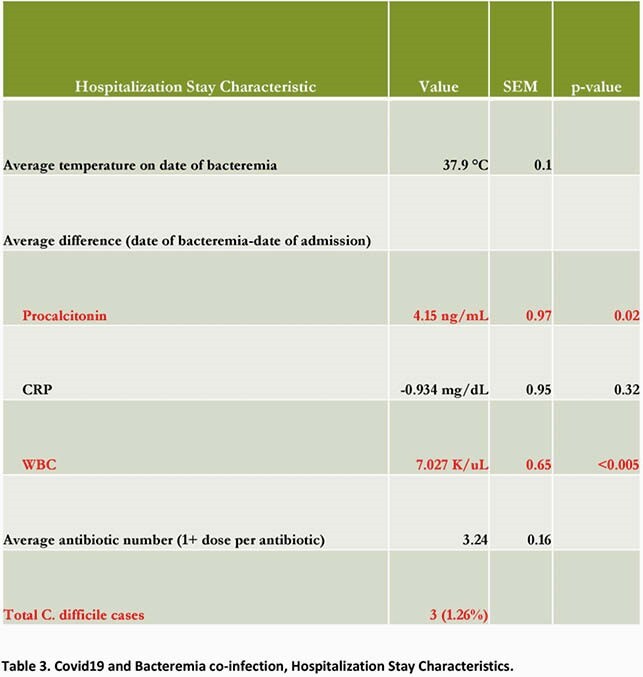

Relevant laboratory parameters for patients with Covid19 and bacteremia co-infection

**Conclusion:**

Patients with Covid19 and bacteremia had high mortality (Figure 2), 53% received immunomodulator therapy, possibly contributing to bacteremia development. With bacteremia increase in WBC and Procalcitonin, not CRP, noted. Most organisms CoNS, likely contaminants, gram positive bacteremias likely from indwelling lines. Only 3 C difficile infections identified. Trends noted in Procalcitonin rise, immunomodulator therapy, and low C difficile infection rates warrant further studies.

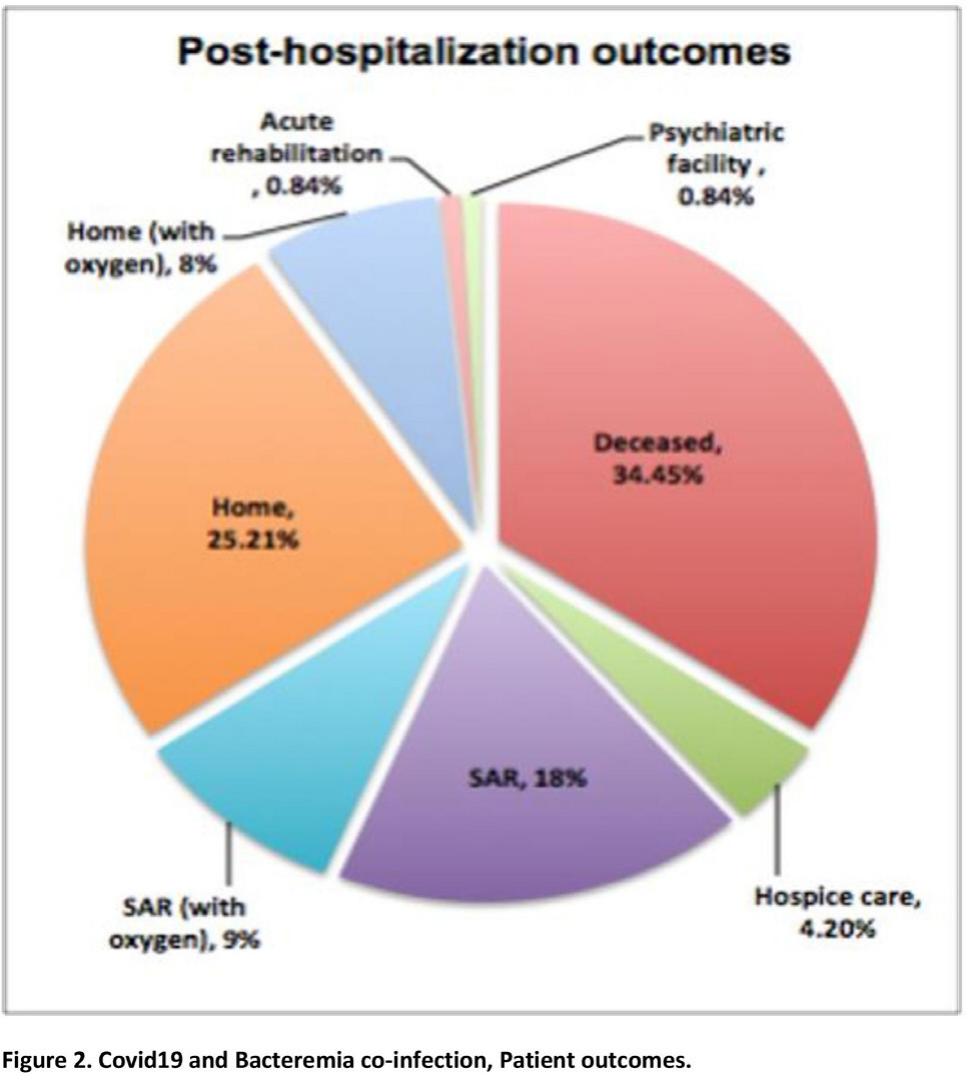

Post-hospitalization Outcomes

**Disclosures:**

**All Authors**: No reported disclosures

**Table 2.**
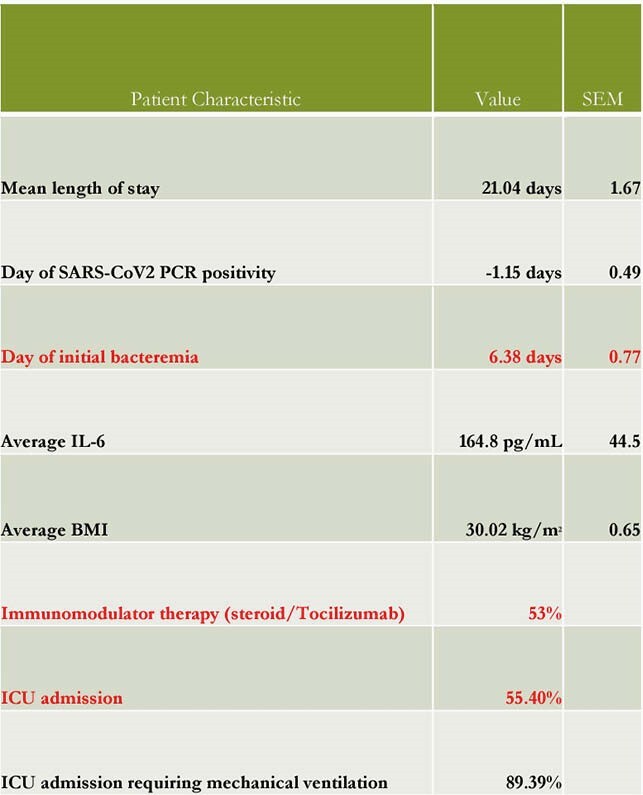
Patient Hospitalization Characteristics

